# Sexual Dimorphism of NADPH Oxidase/H_2_O_2_ System in Rat Thyroid Cells; Effect of Exogenous 17β-Estradiol

**DOI:** 10.3390/ijms19124063

**Published:** 2018-12-15

**Authors:** Jan Stepniak, Andrzej Lewinski, Malgorzata Karbownik-Lewinska

**Affiliations:** 1 Department of Oncological Endocrinology, Medical University of Lodz, 7/9 Zeligowski St., 90-752 Lodz, Poland; jan.stepniak@umed.lodz.pl; 2 Department of Endocrinology and Metabolic Diseases, Medical University of Lodz, 281/289 Rzgowska St., 93-338 Lodz, Poland; alewin@csk.umed.lodz.pl; 3 Polish Mother’s Memorial Hospital—Research Institute, 281/289 Rzgowska St., 93-338 Lodz, Poland

**Keywords:** thyroid, 17β-estradiol, NADPH oxidases, hydrogen peroxide

## Abstract

It has long been observed that females are more susceptible to thyroid diseases than males. Epidemiological and experimental data show that actions of hormonal factors—especially estrogens—may explain such disparity. However, the exact cause and mechanisms of this sexual dimorphism remain so far unknown. Therefore, we aimed at evaluating the effect of 17β-estradiol on the redox balance in thyroids of male and female rats. Expression of nicotinamide adenine dinucleotide phosphate (NADPH) oxidases, i.e., dual oxidase 1 (DUOX1), dual oxidase 2 (DUOX2) and NADPH oxidase 4 (NOX4), and hydrogen peroxide (H_2_O_2_) levels were evaluated in the primary cell cultures derived from thyroid glands of adult male or female Wistar rats. The measurement was made before and after treatment with 17β-estradiol alone or with addition of one of its receptor antagonists. We found that under basal conditions female thyroid cells are exposed to higher concentrations of H_2_O_2_, most likely due to NOX/DUOX enzymes activity. Additionally, exogenous 17β-estradiol stimulated NOX/DUOX expression as well as H_2_O_2_ production, and this effect was mainly mediated through ERα. In conclusion, oxidative processes may constitute mechanisms responsible for sexual dimorphism of thyroid diseases. Exogenous 17β-estradiol may play a crucial pathogenic role in thyroid diseases via oxidative mechanisms, however without any gender differences.

## 1. Introduction

It has long been observed that females are more susceptible to thyroid diseases than males. Both benign and malignant thyroid tumors are 3–4 times more likely to develop in women than in men [1]. Thyroid autoimmune diseases are also highly prevalent, with the highest female-to-male ratio among all autoimmune diseases [2]. The exact cause and mechanisms of this sexual dimorphism remain unknown thus far. In recent years, many factors (such as environmental, dietary or genetic factors) potentially responsible for this phenomenon have been excluded [3,4,5]. On the other hand, evidence from an increasing number of studies show the influence of sex hormones—mainly estrogens—on the pathogenesis of proliferative and autoimmune thyroid diseases.

Estrogens are a class of steroid hormones that play important roles in both reproductive and non-reproductive female and male systems [6]. The primary sites of estrogen synthesis are the gonads (ovaries and testes), although they can also be produced in a number of extra-gonadal organs. Experimental studies show that organs such as the liver, skin or brain can also synthetize a small but significant amount of estrogens [7]. However, estrogen synthesized within these extra-gonadal compartments mostly acts locally at the site of synthesis. The most biologically prevalent and active estrogen is 17β-estradiol. The physiological level of circulating 17β-estradiol is strongly dependent on sex and the reproductive status [8]. 17β-estradiol functions are very broad. It is involved in cellular processes such as proliferation, differentiation, motility, and death, not only in reproductive tissues like breast, endometrium, ovary and testes but also in non-reproductive tissues, including endocrine glands [9]. Cellular signaling of 17β-estradiol is primarily mediated upon the binding on nuclear and extra-nuclear estrogen receptor (ER) alpha and beta (ER-α, ER-β) as well as the G protein-coupled estrogen receptor (GPER) [10,11]. Estrogen receptors have been described in both neoplastic and nonneoplastic thyroid tissues [12,13].

Epidemiological as well as experimental evidence clearly indicates that estrogens are carcinogenic, playing an essential role in thyroid cancer development. There are several mechanisms that are potentially responsible for this phenomenon. Metabolism of estrogens by cytochrome P450s results in the formation of catechols, which in turn can be oxidized to quinone and semiquinone intermediates that react with purine bases of DNA, resulting in depurinating adducts that generate highly mutagenic apurinic sites [14,15]. Interestingly, it was observed that women diagnosed with thyroid cancer have higher levels of estrogen-DNA adducts [16]. Estrogens can also stimulate the proliferation of thyrocytes or more likely thyroid stem cells via receptor-mediated pathway [17,18] or increased thyroid NADPH oxidases stimulation [19]. This last phenomenon is of special importance because it can explain not only the tumorigenic capability of estrogens but also the cause of a higher incidence of autoimmune thyroid diseases in women [20,21]. Furthermore, estrogens are involved in the regulation of angiogenesis and metastasis that are critical for the outcome of thyroid cancer [22]. 

The NADPH oxidase/dual oxidase (NOX/DUOX) family are transmembrane proteins generating reactive oxygen species (ROS) as their primary enzymatic products. They are flavoproteins containing two haem-binding histidines and one NADPH binding site followed by one flavin adenine dinucleotide (FAD) binding site. ROS production is achieved through the removal and transfer of an electron from an NADPH substrate to FAD, then haem, and finally to molecular oxygen, generating superoxide, which is rapidly converted into hydrogen peroxide (H_2_O_2_) [23]. The NADPH oxidases are the major nonmitochondrial sources of ROS in cells. To date, three enzymes belonging to this group have been identified in the thyroid gland, i.e., dual oxidase 1 (DUOX1), dual oxidase 2 (DUOX2) and NADPH oxidase 4 (NOX4). Both DUOX1 and DUOX2 are localized at the apical membrane of thyrocytes and their activation depends on the rise of intracellular calcium levels. The DUOX2 enzyme generates H_2_O_2_, which acts as an electron acceptor for the thyroid hormone biosynthesis. It is the dominant isoenzyme in the thyroid with an expression level that is five times higher than that of DUOX1 [24]. The role of DUOX1 in the thyroid is still unknown. However, it is believed that it could function to overcome the lack of DUOX2 activity, at least under some still undefined circumstances [25].

By contrast to DUOX enzymes, NOX4 is active not only at the plasma membrane but also in different intracellular compartments and organelles, including endoplasmic reticulum, mitochondria and the nucleus. Moreover, its ROS-generating activity is constitutive, depending only on its expression levels [26,27]. The physiological function of H_2_O_2_ produced by NOX4 in the normal thyroid gland is presently unknown. It does not seem to be involved in thyroid hormone biosynthesis (NOX4 knockout animal models do not have thyroid dysfunction) and most likely acts as an intracellular signaling factor. This assumption stems from research data emerging over the last decade which suggests that NOX enzymes-derived ROS play significant roles in signal transduction events. It turns out that activity of H_2_O_2_ can influence a range of cellular events in a manner similar to that seen for traditional second messenger molecules such as cAMP or Ca^2+^ [28]. 

H_2_O_2_ is a non-polar molecule with relatively long half-life able to diffuse across biological membranes. It acts as a messenger molecule by oxidation of critical amino acid residues of proteins, thus affecting their activity. Oxidation of such proteins as phosphatases, transcription factors, ion channels, antioxidant and metabolic enzymes, structural proteins or protein kinases have potential to influence numerous cellular processes like cell proliferation, migration, survival, and death [29]. Specificity of H_2_O_2_ signaling depends on strict control of its sources, cellular localization of production and compartmentalization through the activity of antioxidant or ROS-scavenging systems such as peroxiredoxin enzymes [30,31]. This control can be especially hard in the thyroid gland in which one large quantities of H_2_O_2_ are produced for the needs of hormonogenesis. Impaired cellular oxidant–antioxidant homeostasis in the thyroid can play a significant role in the pathogenesis of thyroid diseases. Stimulation of thyroid NADPH oxidases by 17β-estradiol can lead to increased H_2_O_2_ production and could contribute to the greater incidence of thyroid diseases among women.

It should also be noted that H_2_O_2_ is primarily a toxic compound and its excess can induce DNA oxidation and damage, and consequent mutagenesis and apoptosis. It has been shown that thyroid gland is exposed to higher levels of DNA oxidative damage and spontaneous mutations in comparison to other tissues, which could be due to long-term H_2_O_2_ exposure (the average life span of thyroid cells is 8.5 years in humans [32], and is accordingly shorter—however, still relatively long—in rodents) [33]. It should be stressed that iodine, which is indispensable for thyroid hormone synthesis, may act as either an antioxidant or as a prooxidant, depending on various factors such as its concentration and the presence of other oxidative substances in the environment. In the context of its effects in the thyroid, iodine has been documented to damage macromolecules in the thyroid under experimental conditions [34].

For this perspective, the need for studies emerges concerning gender-specific oxidative stress in order to find out what molecular mechanisms are involved in gender disparity of thyroid diseases. 

The aim of the present study was to compare NADPH/H_2_O_2_ system in rat thyroid between sexes. The next aim was to evaluate the effects of exogenous 17β-estradiol on the level of H_2_O_2_ and expression of enzymes involved in its production in the thyroid of both sexes. Additionally, the involvement of receptor(s) mediating the effects of 17β-estradiol was studied. 

## 2. Results

### 2.1. NOX/DUOX Expression and H_2_O_2_ Level in Primary Cells Derived from Male and Female Rat Thyroids

**Figure 1:**
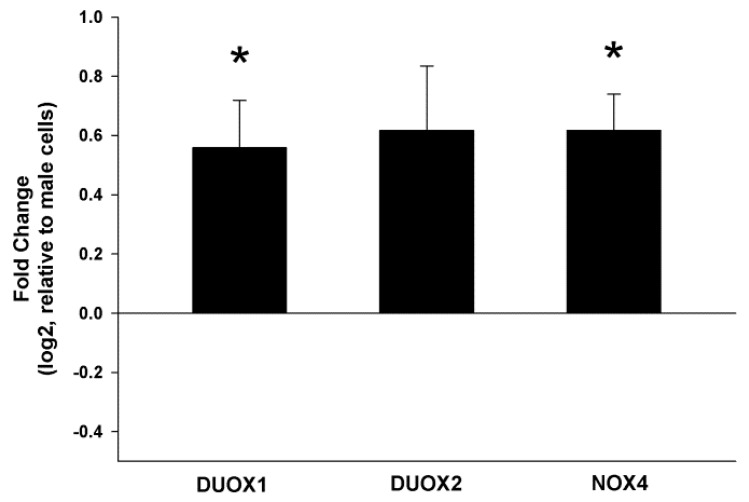
Expression (log2 fold change) of DUOX1, DUOX2 and NOX4 in cells derived from female rat thyroid relative to expression of these genes in cells derived from male rat thyroid. mRNA expression was determined by real-time PCR. Bars represent the mean ± SE of three independent experiments. * *p* < 0.05.

**Figure 2:**
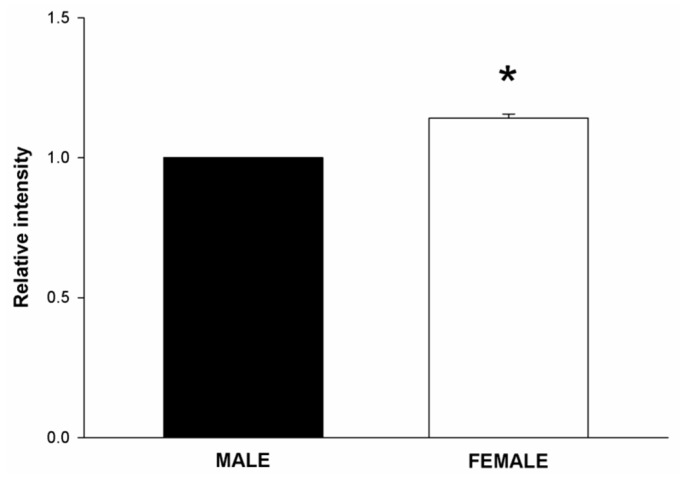
H_2_O_2_ level in cells derived from male and female rat thyroid. The level of H_2_O_2_ was evaluated by Premo^™^ cellular hydrogen peroxide sensor and was expressed relative to the male group. Bars represent the mean ± SE of three independent experiments. * *p* < 0.05.

Total RNA from male and female thyroid primary cells was isolated, and RT-PCR for DUOX1, DUOX2 and NOX4 was performed. We found that the expression of DUOX1 and NOX4 was significantly higher in female thyroid cells as compared to male (1.5 fold, *p* < 0.05). Expression of DUOX2 in female cells was higher but statistically not significant (1.5 fold, *p* = 0.06) (Figure 1). To check whether increased expression of these genes in females translates into increased production of H_2_O_2_, we examined the intracellular level of this molecule. Because chemical probes commonly used to detect intracellular ROS lack specificity for the type of ROS produced, for measuring H_2_O_2_ level in cells we used a recently developed sensor, Orp1-roGFP [35]. We found that female rat thyroid cells produce higher amounts of H_2_O_2_ as compared to male rat thyroid cells (*p* < 0.05) (Figure 2).

### 2.2. Stimulation of NOX/DUOX Expression and H_2_O_2_ Production by 17β-Estradiol

**Figure 3:**
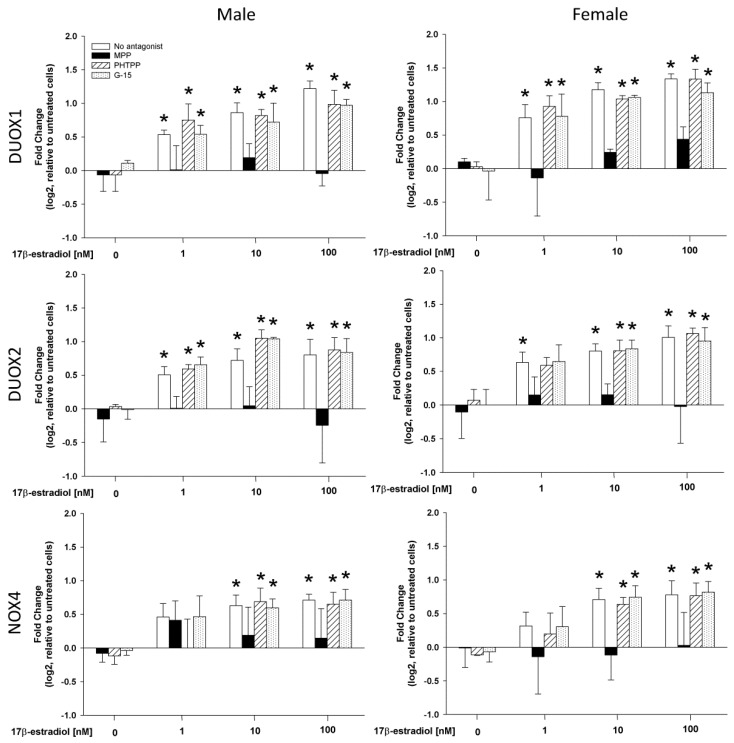
The mRNA expression (log2 fold change) of NOX/DUOX oxidases in cells derived from male and female rat thyroid. Cells were incubated in the presence of 17β-estradiol (0.0 nM, 1.0 nM, 10 nM or 100 nM) alone or with addition of one of the 17β-estradiol receptor antagonists (10 µM). mRNA expression was determined by real-time PCR and was expressed relative to the untreated cells. Bars represent the mean ± SE of three independent experiments. * *p* < 0.05 vs. respective Control antagonist (without 17β-estradiol).

**Figure 4:**
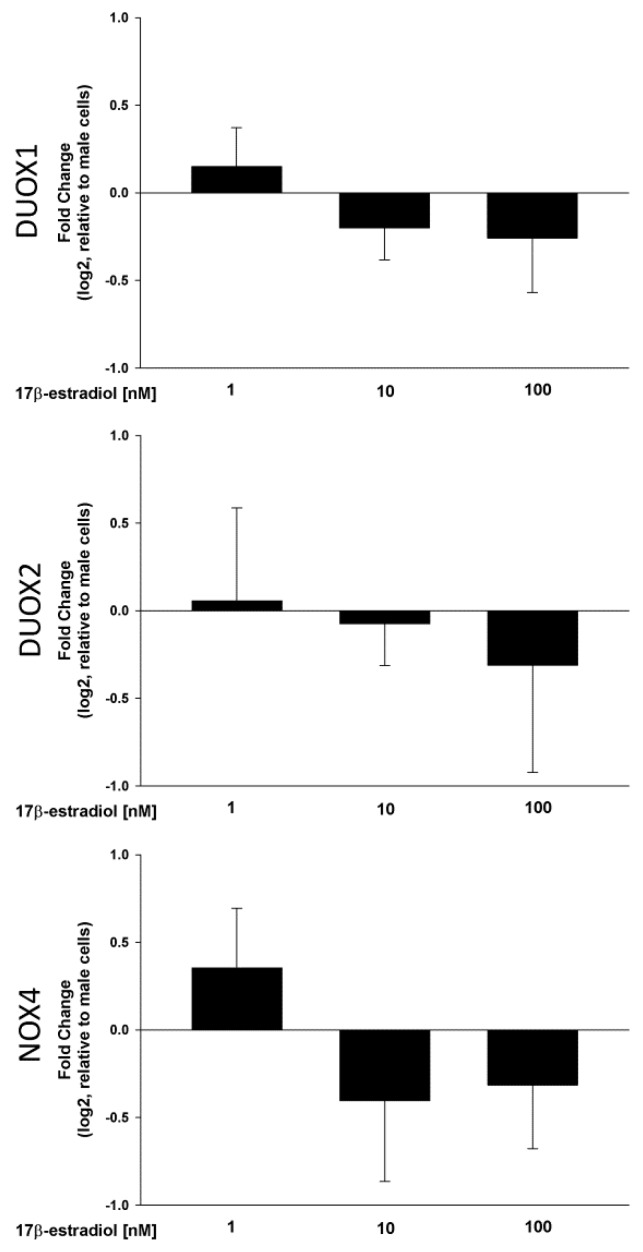
Expression (log2 fold change) of NOX/DUOX oxidases mRNA levels in females. Cells derived from male and female rat thyroid were incubated in the presence of 17β-estradiol (1.0 nM, 10 nM or 100 nM). mRNA expression was determined by real-time PCR and was expressed relative to the male group. Bars represent the mean ± SE of three independent experiments. No significant differences were found.

**Figure 5:**
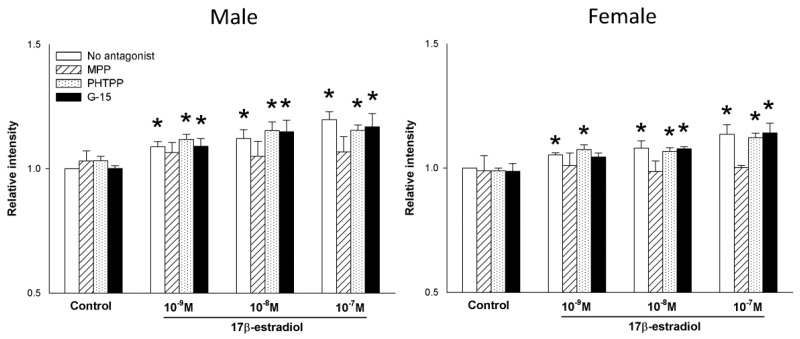
H_2_O_2_ level in cells derived from male and female rat thyroid. Cells were incubated in the presence of 17β-estradiol (0.0 nM, 1.0 nM, 10 nM or 100 nM) alone or with addition of one of the 17β-estradiol receptor antagonists (10 µM). The level of H_2_O_2_ was evaluated by Premo^™^ cellular hydrogen peroxide sensor and was expressed relative to the untreated control group. Bars represent the mean ± SE of three independent experiments. * *p* < 0.05 vs. respective Control antagonist (without 17β-estradiol).

A range of 17β-estradiol concentrations (1.0, 10 and 100 nmol/L) was added to the male and female thyroid cells for 48 h to assess its effect on the expression of DUOX1, DUOX2 and NOX4 as well as H_2_O_2_ production. The analysis of the RT-PCR results showed that 17β-estradiol causes a dose-dependent increase in the expression of the tested genes in cells from both male and female thyroids (Figure 3). In case of DUOX1 and DUOX2 all used concentrations of 17β-estradiol caused increase of expression. In case of NOX4, only two highest concentrations, i.e., 10 and 100 nmol/L, were potent enough to increase expression of that gene. Interestingly, 17β-estradiol was equally potent in its stimulating effects in both sexes (Figure 4). Moreover, the increase in the DUOX1, DUOX2 and NOX4 expression in response to 17β-estradiol is accompanied by an increase in the level of H_2_O_2_. As shown in Figure 5, 17β-estradiol caused a dose-dependent increase of the H_2_O_2_ level.

### 2.3. The Role of Estrogen Receptors in the 17β-Estradiol-Dependent Increase in NOX/DUOX Expression and H_2_O_2_ Production

To evaluate the involvement of receptor(s) mediating the effects of 17β-estradiol, the antagonists specifically for ERα (MPP), for ERβ (PHTPP) or for GPER (G-15) were used. We found that the expression of DUOX1, DUOX2 and NOX4 increase due to the action of 17β-estradiol was not affected by the pre-treatment with either PHTPP or G-15. MPP, however, effectively antagonized the increase of the expression of the tested genes (Figure 4). This ERα antagonist prevented the stimulating effect of all three 17β-estradiol concentrations and maintained the expression of the examined genes at the level of the control group (Figure 4). Blocking of ERα by MPP completely suppressed the stimulating effect of 17β-estradiol on the H_2_O_2_ production (Figure 5). These effects of estrogen receptors antagonists were similar in both sexes.

## 3. Discussion

As was mentioned in the Introduction, the prevalence of different thyroid diseases is higher in women than in men [1,2]. Epidemiological and experimental data show that actions of hormonal factors—especially estrogens—may explain such disparity. Liu et al. demonstrated greater than normal serum levels of estrogen, follicle-stimulating hormone and luteinizing hormone (and their receptors) in thyroid tissue in patients with various types of thyroid neoplasms [13], which strongly suggest that thyroid neoplasms might be sex hormone-dependent. Experimental studies show that 17β-estradiol is a potent stimulator of both human benign and malignant thyroid cells [36,37]. This can explain the fact that incidence of thyroid cancer increases in females with the onset of puberty and decreases after menopause [38]. Even in males it was found that higher 17β-estradiol to testosterone ratios were significantly associated with thyroid autoimmune diseases [39]. On the other hand, studies show that hysterectomy with oophorectomy in women—a procedure which reduces the production of estrogens—does not decrease, but actually increases thyroid cancer risk [40]. This effect, however, can be a result of factors other than estrogens [19]. 

17β-estradiol can exert its action by many pathways, therefore determination of the exact mechanism by which it negatively affects the thyroid is difficult. However, recent experimental studies suggest that ROS produced as an effect of 17β-estradiol action can play a critical role in sexual dimorphism of thyroid diseases [19,41,42]. It is hypothesized that oxidative stress may underlie both proliferative and autoimmune thyroid diseases. In fact, in this study we found that expression of DUOX1 and NOX4—major nonmitochondrial sources of ROS—is significantly higher in cells derived from female thyroid than in cells derived from male thyroid. This is consistent with the results of previous experimental studies in which the existence of a sexual dimorphism in the expression of NOX4 [19] and DUOX1 [43] was found, and can suggest a role of estrogens in the control of the expression of these genes. Moreover, it was demonstrated that NOX4 are overexpressed in thyroid cancers, linking this H_2_O_2_-generating system to cancer pathogenesis [44]. Overexpression of DUOX1 and DUOX2 in thyroid cancers was also reported, however, the results are inconsistent [45,46]. Upregulation of DUOX1 expression can also underlie or contribute to autoimmune thyroid diseases [47]. 

It should be stressed that mRNA levels may not necessarily reflect protein levels and ROS formation. It was shown that NOX4 is regulated at both transcriptional and post-transcriptional levels, and the steady state level of NOX4 mRNA does not accurately reflect NOX4 protein abundance and functions [48]. For these reasons, in these studies we also measured the cellular level of H_2_O_2_—primary enzymatic product of NOX/DUOX enzymes. It turned out that the level of H_2_O_2_ also was higher in cells derived from female thyroids than in cells derived from male thyroids. Chronic exposition of female thyroid to higher levels of H_2_O_2_ can potentially cause many adverse effects. H_2_O_2_ acting as oxidizing agent can lead to damage of macromolecules such as proteins, lipids and nucleic acids [49,50,51,52]. Moreover, H_2_O_2_ acting as a signaling molecule can cause higher cell proliferation rate and inhibition of apoptosis [53]. All of the above might be associated with increased risk of thyroid disorders. 

In present work NOX/DUOX expression level was upregulated by exogenous 17β-estradiol in a dose-dependent manner which was followed by an increase in H_2_O_2_ levels. Interestingly, 17β-estradiol was equally potent in its stimulatory effect on cells derived from male and female thyroid glands. This phenomenon underlines the relevance of the hormone effect rather than of other sex-specific factors. Experimental studies on human thyroid tumor cells show that 17β-estradiol have well-described dose-dependent effect on thyrocyte proliferation [54]. This dose-dependent effect of 17β-estradiol has been well confirmed concerning its influence on oxidative processes in the present study. Stimulation of H_2_O_2_ production can be a crucial point of the process of proliferation, since this molecule can activate signaling cascades via phosphatidylinositol-3-kinase (PI3K) [55]. PI3K signaling cascade is one of the main activation pathways implicated in the pathogenesis of thyroid proliferative and neoplastic disorders [56]. It is worth mentioning that circulating estrogens increased thyroid follicular cells proliferation via PI3K activation in the in vivo model [43].

The effects of 17β-estradiol on target tissues are primarily mediated by two nuclear receptors i.e., ERα and ERβ, and membrane receptor GPER. Although both types of ER (i.e., intracellular ER-α/ER-β and membrane GPER) transduce 17β-estradiol signals, ER-α/ER-β initiate the biological events after a time-lag of at least 2 h, while the cell membrane ERs trigger an intracellular signaling cascade response in seconds [57]. ERα is well characterized as a mediator of cell proliferation, especially in breast cancer cells. In contrast, ERβ is associated with apoptosis and inhibits ERα-mediated proliferation in many cell types [58]. In recent years, a new class of receptors known as estrogen-related receptors (EERs) gained importance in the context of endocrine tumor diseases. EERs are nuclear receptors with high structural homology to ERs. Despite the fact that estradiol and other natural estrogens are not ERRs ligands, they can actively influence the estrogenic response by substituting for ERs activities [59], and can play important roles in mitochondrial activity [60]. In previous experimental studies in rodents no significant differences between males and females in ERα, ERβ, and GPER expression were found [19,43]. It was found, however, that ERα was expressed at much higher levels than the other two receptors, suggesting that ERα is the main estrogen receptor in the thyroid [43]. This is in line with the results of the present study showing that only MPP—selective ERα antagonist—has a significant impact on NOX/DUOX expression and H_2_O_2_ production. These results suggest that the effects of 17β-estradiol on H_2_O_2_ production in thyroid are predominantly mediated through ERα. However, the action of MPP was similar in both sexes. Thus, the last observation does not confirm that receptor mechanisms via ERα in the thyroid are involved in the phenomenon of sexual dimorphism. Both ERβ and GPER antagonists (PHPTT and G-15 respectively) did not affect the NOX/DUOX expression and H_2_O_2_ production. Previous studies on human thyroid cancer cells demonstrated that the agonist of ER*α* (PPT) enhanced cell proliferation and growth while the agonist of ER*β* (DPN) acted as an inhibiting force [61]. These findings, together with our results, further support the role of H_2_O_2_, produced in response to 17β-estradiol, in the process of thyroid cells proliferation. 

In conclusion, the results of the present study confirm and extend earlier findings on the existence of a gender disparity in the thyroid cells. Our study has shown that female thyroid cells are exposed to higher concentrations of H_2_O_2_, most likely due to higher NOX/DUOX enzymes activity. Additionally, exogenous 17β-estradiol have a major impact on the redox state of thyroid cells through the stimulation of NOX/DUOX expression and, consequently, stimulation of H_2_O_2_ production. Our data have also shown that this effect is mainly mediated through ERα. Taken together, these results provide evidence that oxidative processes, at least NADPH oxidases/H_2_O_2_, may constitute mechanisms responsible for sexual dimorphism of thyroid diseases and that exogenous 17β-estradiol may play a crucial pathogenic role in thyroid diseases. Thus, our results may provide insights into the molecular mechanism underlying the epidemiological data that show a higher prevalence of thyroid diseases in females than in males.

## 4. Materials and Methods

### 4.1. Materials 

Unless otherwise stated, all reagents were purchased from Sigma-Aldrich, Saint Louis, MO, USA.

### 4.2. Cell Culture

Thyroid glands were collected from adult male (*n* = 50) or female (*n* = 50) Wistar rats that had been euthanized by sodium pentobarbital overdose. The glands were freed from adherent connective tissue and minced into small fragments using a sterile razor blade in a cell culture hood. After one wash in Hanks’ balanced salt solution (HBSS), the tissue fragments were digested in 1 mg/mL collagenase IV and 2.5 mg/mL trypsin in HBSS for 40 min at 37 °C. The digested material was filtered through a nylon mesh (100 μm; Sigma-Aldrich, Saint Louis, MO, USA) to remove undigested tissue fragments. The cells were washed twice with HBSS and centrifuged at 1000× *g* for 5 min and then seeded to a density of 0.05 × 10^6^ cells in 0.5 mL of culture medium per well in 24-well plates (Nunc UpCell 24 Multidish; Thermo Fisher Scientific, Waltham, MA, USA). Culture medium consisted of Coon’s modified Ham’s F-12 medium (Biochrom, Berlin, Germany) supplemented with 10% calf serum, 2 mM glutamine, a five-hormone mixture (1 mIU/mL thyroid-stimulating hormone, 10 ng/mL somatostatin, 10 μg/mL insulin, 1 nM hydrocortisone, and 5 μg/mL transferrin), 100 IU/mL penicillin–streptomycin, and 2.5 µg/mL amphotericin B. Immediately after plating, cells were transduced with Orp1-roGFP redox sensor. Cells were maintained in culture at 37 °C in air:CO_2_ (95:5%) atmosphere. 17β-estradiol and/or its antagonist’s treatments were added after 48 h of incubation. 

### 4.3. Cell Treatment

Twenty-four hours before the experiments, the medium was removed and replaced with a medium without phenol red supplemented with 0.5% calf serum and a five-hormone mixture. Male or female cells were treated with 17β-estradiol (0.0 nM, 1.0 nM, 10 nM or 100 nM) alone or with addition of one of the 17β-estradiol receptor antagonists, i.e., 10 µM of ERα antagonist MPP (Tocris Bioscience, Bristol, UK), 10 µM of ERβ antagonist PHTPP (Tocris Bioscience) or 10 µM of GPER antagonist G-15 (Tocris Bioscience). Stock solutions of 17β-estradiol (1.0 µM, 10 µM and 100 µM) and its receptor antagonists (10 mM) was prepared in dimethyl sulfoxide (DMSO) and stored at −20 °C. Control cells were treated with DMSO (final conc. 0.1%). The treated cells were incubated for another 24 h in the starvation medium. 

### 4.4. Cell Viability Assay

Cell viability was determined by performing a trypan blue exclusion assay. Cell suspension was stained with 0.4% trypan blue solution (1:1 mixture) after which the number of viable cells were counted using a microscope. 

### 4.5. Evaluation of H_2_O_2_ Level

Cellular H_2_O_2_ levels were quantified using the Premo^™^ cellular hydrogen peroxide sensor Orp1-roGFP (Life Technologies, Carlsbad, CA, USA), according to the manufacturer’s instructions. Plasmid DNA encoding Orp1-roGFP was delivered into the cell via baculovirus transduction. Cells were transduced with Orp1-roGFP redox sensor immediately after plating at a multiplicity of infection of 200 pfu. 

After 24 h of incubation with 17β-estradiol and/or its antagonists, fluorescence was measured in a Synergy H1 Microplate Reader (BioTek, Winooski, VT, USA) using excitation at 400 nm and 488 nm, and emission at 515 nm. Fluorescence intensity values were used to calculate 400/488 nm excitation ratios. The results are expressed relative to excitation ratio in untreated cells.

### 4.6. mRNA Analysis by qRT-PCR

Total RNA was extracted from cells using the GenElute™ Mammalian Total RNA Miniprep Kit (Sigma-Aldrich) according to the manufacturer’s protocol. RNA concentration and purity were measured using the NanoDrop™ N-1000 spectrophotometer (Nanodrop Tech, Wilmington, DE, USA). Reverse transcription was performed by using High-Capacity cDNA Reverse Transcription Kit (Life Technologies). After that real-time PCR was performed on the ABI PRISM^®^ 7500 Sequence Detection System (Applied Biosystems, Waltham, MA, USA) by using *Taq*Man^®^ Universal PCR Master Mix (Life Technologies) and *Taq*Man^®^ Gene Expression Assays probe and primer mix (Life Technologies) according to the manufacturers’ specification. The Assay Identification numbers were: *DUOX1*-Rn00596688_m1; *DUOX2*-Rn00666512_m1; *NOX4*-Rn00585380_m1; *GAPDH*-Rn01775763_g1. An analysis of relative gene expression data was performed, using the 2^−ΔΔ*C*T^ method on an ABI PRISM^®^7500 Sequence Detection System Software (Applied Biosystems). Expression of *DUOX1*, *DUOX2* and *NOX4* in treated cells was quantified relative to the expression of these genes in untreated cells. The fold change in studied gene expression, normalized to endogenous control, was calculated using formula: RQ = 2^−ΔΔ*C*T^. Results was presented as a log2 of fold changes (RQ) value. 

### 4.7. Statistical Analysis

Statistical analysis was performed on all data sets, and significant differences were determined by *t* test or by the one-way analysis of variance test followed by the Newman-Keuls multiple comparison test. Results of the qRT-PCR was presented as a log2 of fold changes (RQ) value relative to the male or untreated control group. The results are presented as mean values ± S.E. from a three independent experiments. A level of *p* < 0.05 was considered to be significant.
